# Intraoperative Fluorescein Sodium in Pediatric Neurosurgery: A Preliminary Case Series from a Singapore Children’s Hospital

**DOI:** 10.3390/neurosci4010007

**Published:** 2023-02-13

**Authors:** Audrey J. L. Tan, Min Li Tey, Wan Tew Seow, David C. Y. Low, Kenneth T. E. Chang, Lee Ping Ng, Wen Shen Looi, Ru Xin Wong, Enrica E. K. Tan, Sharon Y. Y. Low

**Affiliations:** 1 Department of Neurosurgery, National Neuroscience Institute, 11 Jalan Tan Tock Seng, Singapore 308433, Singapore; 2 Neurosurgical Service, KK Women’s and Children’s Hospital, 100 Bukit Timah Road, Singapore 229899, Singapore; 3 SingHealth Duke-NUS Neuroscience Academic Clinical Program, 11 Jalan Tan Tock Seng, Singapore 308433, Singapore; 4 Department of Pathology and Laboratory Medicine, KK Women’s and Children’s Hospital, 100 Bukit Timah Road, Singapore 229899, Singapore; 5 Department of Radiation Oncology, National Cancer Centre, 11 Hospital Drive, Singapore 169610, Singapore; 6 Paediatric Haematology/Oncology Service, KK Women’s and Children’s Hospital, 100 Bukit Timah Road, Singapore 229899, Singapore

**Keywords:** intraoperative fluorescence, pediatric brain tumors, neurosurgical adjunct

## Abstract

(1) Background: Fluorescein sodium (Na-Fl) has been described as a safe and useful neurosurgical adjunct in adult neurooncology. However, its use has yet to be fully established in children. We designed a study to investigate the use of intraoperative Na-Fl in pediatric brain tumor surgery. (2) Methods: This is a single-institution study for pediatric brain tumor patients managed by the Neurosurgical Service, KK Women’s and Children’s Hospital. Inclusion criteria consists of patients undergoing surgery for suspected brain tumors from 3 to 19 years old. A predefined intravenous dose of 2 mg/kg of 10% Na-Fl is administered per patient. Following craniotomy, surgery is performed under alternating white light and YELLOW-560 nm filter illumination. (3) Results: A total of 21 patients with suspected brain tumours were included. Median age was 12.1 years old. For three patients (14.3%), there was no significant Na-Fl fluorescence detected and their final histologies reported a cavernoma and two radiation-induced high grade gliomas. The remaining patients (85.7%) had adequate intraoperative fluorescence for their lesions. No adverse side effects were encountered with the use of Na-Fl. (4) Conclusions: Preliminary findings demonstrate the safe and efficacious use of intraoperative Na-Fl for brain tumors as a neurosurgical adjunct in our pediatric patients.

## 1. Introduction

Brain tumors are the most common solid tumors in the pediatric population and, unfortunately, the leading cause of childhood cancer-related deaths [1,2]. Presently, tissue diagnosis via biopsy or resection is paramount for diagnosis and molecular studies that will impact subsequent treatment options. Nonetheless, accurate tissue sampling for brain biopsy and achieving a good extent of resection (EOR) for large tumors can be technically challenging. In recent years, the use of intraoperative fluorescence has been accepted as a safe and useful neurosurgical adjunct in the field of neurooncology [1,2,3]. One such example is fluorescein sodium (Na-Fl)—a sodium salt and an organic fluorescent dye that has been extensively investigated in adult high-grade gliomas (HGG) [4,5,6]. Na-Fl disseminates through a disruption in the blood–brain barrier (BBB) and accumulates in the extracellular space of brain tumors [7]. However, its use for the same purpose is comparatively less established in children. At the time of this writing, most of these studies are based in overseas centers and limited to case series or case reports [7,8,9,10]. Overall, there is a paucity of data with regards to the utility of Na-Fl for pediatric brain tumor surgery from our region of Southeast Asia. To address its safety and efficacy for our local population, we designed a study to investigate the use of intraoperative Na-Fl in pediatric brain tumor surgery. Concurrently, the reliability and technical aspects of its use are evaluated in corroboration with current literature.

## 2. Materials and Methods

### 2.1. Study Design and Patient Selection

This is an ethics-approved, retrospective study for pediatric brain tumor patients managed by the Neurosurgical Service, KK Women’s and Children’s Hospital (SingHealth CIRB Ref: 2014/2079). Inclusion criteria involves patients undergoing surgery for either suspected or known intracranial tumors from 3 to 19 years old. Patients less than 3 years old, with known allergic reactions to Na-Fl, diagnosed with significant systemic comorbidities such as cardiac, hepatic or renal dysfunction, and or hyperreactivity towards contrast agents are excluded. 

### 2.2. Outline of Operative Procedure and Perioperative Management

At our institution, all patients with suspected brain tumors undergo a standard preoperative cranial magnetic resonance imaging (MRI) scan. The StealthStation^TM^ S8 Surgical Navigation System (Medtronic, Minneapolis, MN, USA) is used for preoperative planning and intraoperative neuronavigation. Depending on the location of the tumor, intraoperative neuromonitoring may be applied at the discretion of the neurosurgeon in charge. Following induction of general anesthesia, a predefined dose of 2 mg/kg of 10% Na-Fl 10% is administered intravenously, with simultaneous monitoring of the patient’s vital signs and clinical status. Close monitoring of the patient is continued throughout the surgery, in particular looking out for cutaneous and/or physiological manifestations of an adverse drug reaction. With regards to the brain tumor surgeries, patients are approached using standard microsurgical techniques for tumour resection or stereotaxy-based biopsies. For this study, either the Zeiss KINEVO 900 or Zeiss OPMI Pentero 900 (both from Carl Zeiss, Jena, Germany) surgical microscopes equipped with a YELLOW-560 nm filter are used. Once the dura is exposed, the YELLOW-560 nm filter is turned on to check for Na-Fl staining as a positive control to ensure the dye is working. Dura opening is performed under standard white light and subsequently switched to the YELLOW-560 nm filter during the course of the surgery as required. Briefly, a surgical plan created in neuronavigation console is then used, and sulcal dissection to the lesion of interest takes place. Once the lesion is localized, resection is made under alternating white light and the YELLOW-560 nm filter illumination. A routine postoperative MRI scan is performed the following day to assess the EOR. Separately, for the stereotactic biopsy procedures, we adapted a similar technique as described in the literature [11,12,13]. Here, an intralesional target point is planned via the neuronavigation console. Typically, the target point is chosen within the core of of the lesion. Next, the safest trajectory to the target is selected to avoid critical neurovascular and eloquent structures. Upon tissue sampling, the specimens are physically assessed under the YELLOW 560 nm filter. Fluorescent samples are then correlated with the frozen section report by the on-site pathologist. A computed tomographic (CT) brain scan is ordered after the biopsy to exclude significant bleeding within 24 h. For the purposes of this study, we verified the efficacy of Na-Fl via a feedback evaluation form by the operating neurosurgeons. 

### 2.3. Patient Demographics, Radiological Features, and Variables of Interest

Individual patients’ clinical history, operative notes and radiological records are reviewed to identify variables such as age of diagnosis, gender, clinical status, pre- and post-operative imaging characteristics, perioperative complications and timing of surgery. In addition, the histopathological results of biopsied tissue are correlated with the diagnosis according to the relevant WHO classification within the recruitment time period [14,15]. Statistical analyses are generated using GraphPad Prism version 9.4.1 for Windows (GraphPad Software, La Jolla, CA, USA). As this study has a limited population, descriptive statistics are reported. This includes mean with standard deviation for continuous data, and frequency and percentage for categorical data. 

## 3. Results

### 3.1. Overview of Study Population and Its Characteristics

**Figure 1:**
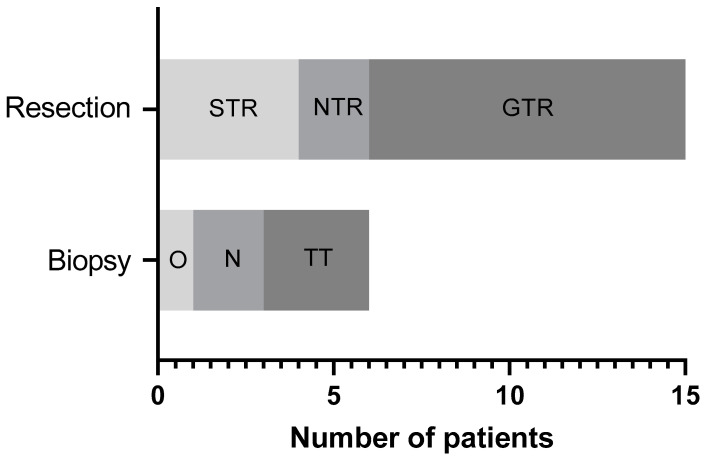
Graph depicting procedure types used in study. Abbreviations: STR = subtotal resection; NTR = near total resection; GTR = gross total resection; O = open; N = needle; TT = transtubular.

From 01 January 2019 to 30 November 2022, a total of 21 patients (13 males and 8 females) radiologically diagnosed with suspected brain tumours were included in this study. Median age of the cohort was 12.1 years old (±5.3 years, range 3 to 19 years old). Fifteen patients (71.4%) underwent resection (8 GTR, 2 NTR and 5 STR) and 6 (28.6%) had tissue biopsies (one open biopsy, two using stereotactic brain needle biopsies and three via the NICO Brainpath^®^ transtubular system). Figure 1 summarizes the types of procedures used in this study. For the purposes of this study, we defined <5% of tumor remnant as achievement of gross total resection (GTR), <10% tumor remnant as near total resection (NTR) and ≥30% tumor remnant as subtotal resection (STR). Concurrent neurosurgical adjuncts implemented included diffusion tensor imaging (DTI) for 13 patients (61.9%), intraoperative neuromonitoring (IONM) for three patients (14.3%) and the intraoperative MRI operating theatre (iMRI OT) for one patient (4.8%). Seven patients (33.3%) had Na-Fl as their only operative adjunct. All patients did not have any acute or delayed adverse effects from Na-Fl. For three patients (14.3%), there was no significant Na-Fl fluorescence detected intraoperatively. Final histology reported a cavernoma and two HGGs that were likely radiation-induced. An overview of the patients’ demographics and variables is featured in Table 1.

### 3.2. Evaluation of Na-Fl as an Intraoperative Adjunct

Three pediatric neurosurgeons from the Neurosurgical Unit, KK Women’s and Children’s Hospital, participated in this study. All of them agreed the fluorophore was easy to firstly prepare (that is, via a straightforward dilution of 2 mg/kg in sterile water) and next to administer intravenously as a bolus dose through a peripheral catheter. Overall, this process only required a few minutes and did not delay the start time of each surgery. We noted that all patients showed a self-limiting neon yellow urine discoloration for up to 12 h after surgery. This is consistent with the known mechanism of Na-Fl, whereby it undergoes hepatic metabolism and subsequently is excreted in the urine [16,17]. Following that, most of the histologically proven tumors demonstrated adequate fluorescence at the start of the surgery, allowing the operating neurosurgeon to visualize tumor boundaries in real time for those requiring resection. With regards to the biopsy cases for tumors located in deeper regions such as the thalamus and brainstem, Na-Fl was useful in confirming our samples were lesional. This is similar to what others have reported in the literature [10,18].

**Figure 2:**
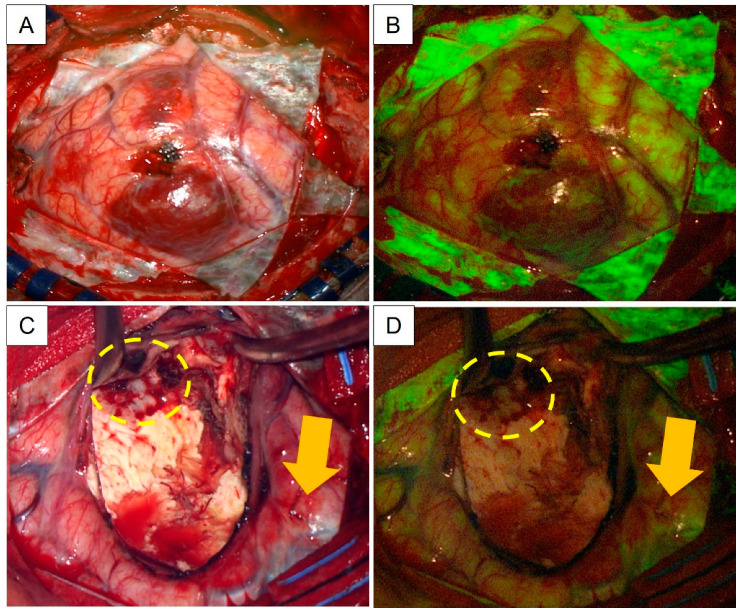
Intraoperative photos taken during resection of a parietal HGG. (**A**,**B**) Visualization of the tumor at the start of surgery under white light versus YELLOW-560 nm filter. Of note, there is good localization of the lobar tumor by the Na-Fl fluorescence. (**C**,**D**) Surgical cavity at the end of resection under white light versus YELLOW 560 nm filter. There was a small, greyish area that was uncertain for remnant tumor under white light (marked out by the yellow circle in dashed lines). However, it did not show fluorescence under the YELLOW-560 nm filter. Additionally, there was a superficial area of fluorescence (yellow arrows) that was outside of the resection margin that did not correlate with the preoperative MRI scans.

For the only non-neoplastic lesion in our cohort, Na-Fl proved to be a relevant negative. However, we had three brain tumor cases that did not show significant fluorescence. Although there were no technical difficulties switching the illumination between white light and fluorescence on the microscope handle, we noted the following during the course of surgery. Firstly, if a vascular tumor was encountered, the fluorescence was not able to properly delineate the brain–tumor interface due to blood products. Under such circumstances, it was challenging to constantly switch between white light and the YELLOW-560 nm filter during active hemostasis for visualization purposes. Another pertinent observation was the difficulty in differentiating between remnant tumor versus gliotic brain towards the end of the resection, especially for large lesions with radiologically proven perilesional oedema. Nonetheless, postoperative MRI brain scans performed within 48 h after resection reported adequate resection margins that correlated with initial surgical aims. Figure 2 shows an example of our experience with intraoperative Na-Fl for the resection of a hemorrhagic parietal HGG.

### 3.3. Illustrative Cases of Interest: Radiation-Induced Gliomas

**Figure 3:**
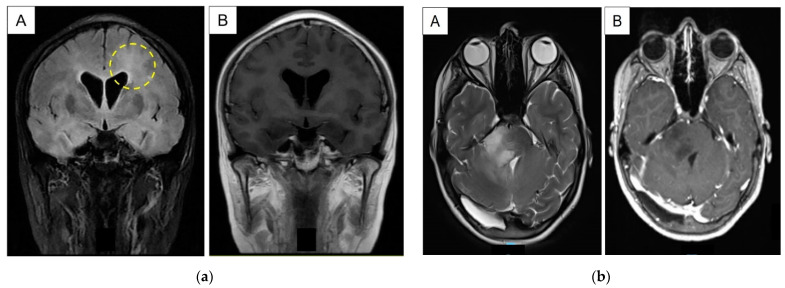
(**a**) Representative MRI coronal images in fluid attenuated inversion recovery (FLAIR) and post-contrast T1-weighted sequences, labelled (**A**) and (**B**) respectively. The decision was made for the biopsy to be taken adjacent to the left frontal horn (marked out by yellow circle in dashed lines) where there were more FLAIR-attenuated changes. (**b**) Representative MRI axial images in T2-weighted and post-contrast T1-weighted sequences, labelled (**A**) and (**B**) respectively. The patient underwent stereotactic needle biopsy of the right pontine lesion.

Interestingly, there were two patients with secondary HGGs that did not demonstrate fluorescence in our study. The first case was a previously well female who was diagnosed with a Group 3 medulloblastoma when she was 12 years old. She underwent gross total resection of her tumor followed by adjuvant craniospinal irradiation with tumor bed boost (54 Gy in 30 fractions) and systemic chemotherapy (CHP Protocol). The patient remained in complete remission without radiological evidence of disease recurrence in her follow-up scans. However, she presented 7 years later with worsening headaches. Repeat MRI of her neuroaxis reported bilateral diffuse patchy T2-weighted hyperintensities in the periventricular and subcortical white matter of bilateral cerebral and cerebellar hemispheres, with marked diffuse cerebral oedema, increased prominence, and new long segments of abnormal signal in the cervical cord. Follow-up blood and CSF via lumbar puncture investigations for infective causes were unremarkable. The decision was made to perform a biopsy to ascertain diagnosis. The second case was a 14-year-old female with a background of posterior fossa ependymoma that underwent gross total resection, followed by adjuvant radiotherapy (54 Gy in 30 fractions) when she was 14 months old in an overseas institution. She was subsequently referred to us for continuity of care. Surveillance scans demonstrated an ill-defined right pontine lesion that was progressively enlarging. Similar to the previous case, a biopsy was recommended to exclude malignancy. See Figure 3 for the representative pictorials of both cases.

## 4. Discussion

### 4.1. Surgery for Pediatric Brain Tumors: Technical Challenges

Pediatric brain tumors are a heterogenous group of neoplasms that differ biologically from their adult counterparts [19]. There is often variability of clinical presentations and considerable delays in their diagnoses [20]. Under such circumstances, the role of the pediatric neurosurgeon is paramount to either provide lifesaving intervention via cytoreduction if there is a significant mass lesion, or in event of deep-seated or multiple brain lesions, to perform a diagnostic brain biopsy [21]. Here, the technical challenges include firstly, ascertaining that the extent of resection is adequate and next, attaining accurate tissue sampling for diagnosis to guide the next step of treatment. Furthermore, current neuronavigation systems that rely on preoperative images often encounter the issue of brain shift, a recognized phenomenon that may cause inaccurate patient-to-image mapping as surgery progresses [22]. Other established adjuncts in pediatric neurosurgery, such as the intraoperative MRI and intraoperative ultrasound, have their own drawbacks, such as high costs, additional technical expertise and so forth [23].

Broadly speaking, the basis for fluorescence-guided surgery in children is largely extrapolated from what is demonstrated in adult malignant gliomas—that standard white light operating microscope may not be able to fully delineate intrinsic tumors from surrounding parenchyma [24]. Fluorescence enables resection to be effectively facilitated via demarcating tumor margins from normal brain tissue during surgery [25,26]. This important ability to delineate the brain–tumor interface and increase tumor visibility in real time circumvents the concerns of inevitable brain shift and can aid resection definitively [27]. Additionally, we are aware that non-diagnostic yields can be up to 13% in stereotactic brain needle biopsies [28]. However, recent studies report that ex vivo confirmation of Na-Fl fluorescence from biopsy samples has a high positive predictive value [12,21].

### 4.2. Fluorescent Dyes for Brain Tumor Surgery: An Overview

To date, there are a few intraoperative fluorescence dyes well established in adult brain tumor surgery. Examples include 5-aminolevulinic acid (5-ALA), indocyanine green (ICG) and Na-Fl [2,26,29]. Nonetheless, 5-ALA in children remains an off-label use. Several case reports on fluorescence-guided brain tumor surgery use in children have been published, yet no prospective study has been conducted [24,30]. Furthermore, previous studies have observed significant correlation between higher postoperative alanine aminotransferase values and younger age [31]. At this juncture, there is no available pharmacokinetic data for 5-ALA in children [24]. Overall, there is sparse data underlying the mechanisms of 5-ALA uptake in various types of brain tumors in children [31]. Following that, ICG is a well-studied water-soluble tricarbocyanine fluorescent dye [32]. Specifically in neoplasms, there is a difference in retention of ICG between tumor and normal tissue due to increased vascular permeability and impaired lymphatic drainage in the tumor microenvironment [33]. Consequently, small ICG molecules accumulate passively in the tumor due to this enhanced permeability and retention effect, thereby providing some tumor contrast for intraoperative real-time tumor recognition [32]. Its efficacy has been safely reported in children undergoing surgery for solid tumors [32]. However, similar to the issues faced for the use of 5-ALA in children, little is known about the applications of ICG and ideal dosing for pediatric brain tumor surgery.

Separately, Na-Fl is an organic fluorescent dye that has been safely used in humans for many years, especially in ophthalmology for retinal angiography [34,35]. It emits fluorescent radiation at wavelengths between 500 and 550 nm, with a peak excitation at 490 nm. It acts as a marker of blood–brain barrier damage by accumulating in extravascular spaces where the blood–brain barrier is disrupted [36,37,38,39]. Accordingly, adult-based Na-Fl studies report that high-grade glioma margins correlate well with gadolinium-based MRI results [36,40]. The key advantage is its utility in the immediate improved visualization of brain tumor tissue and thus improvement of the extent of resection [41,42,43]. Additionally, there are studies that incorporate the use of diffusion tensor imaging (DTI) with Na-Fl to facilitate the maximal resection of adult malignant gliomas in eloquent brain regions [44]. Although adequately established in adult brain tumors, the efficacy of Na-Fl in children remains equivocal [7]. In addition, the optimal dose of Na-Fl for brain tumor surgery in children should be determined, as higher doses may cause side effects such as staining of skin and mucosa and occasionally anaphylaxis [9,30,35]. Following that, the ideal timing of administration also needs to be clarified to avoid nonspecific extravasation of fluorescein into regions of perifocal oedema [17]. A balance must be struck between dosing that creates an increased proportion of unbound Na-Fl that is more likely to unspecifically stain a normal brain but more readily stain tumors [1]. Separately, some authors have also mentioned that fluorescence-guided resection techniques can be limited by the extent of vascular permeability in tumor regions, resulting in the failure to stain the full volume of tumor [1]. Nonetheless, there is promising evidence that Na-Fl is safe and versatile as a neurosurgical adjunct for different types of pediatric brain tumors [8]. 

As this is the first time Na-Fl is being used for this purpose in our institution, the decision to adopt a lower dose of 2 mg/kg of Na-Fl from the literature was for safety concerns [8,13]. We are cautious, as there have been reports of major adverse drug reactions (ADR) as the dose increases [45,46,47]. Given that the use of intravenous Na-Fl has not been previously reported in our part of the world, we are uncertain of its potential side effects on our patients. This is relevant as there is a known prevalence of atopic disorders, including drug allergies, in Singaporean children that may lead to life-threatening ADR [48,49]. Common side effects include of Na-Fl staining in the skin, sclera, mucous membrane and bodily fluids. These reactions are usually mild, self-limiting and dose-dependent [45]. Rarely, serious ADRs such as sickle crisis, hemolytic anemia, vasovagal reactions, and cardiac or respiratory compromise can occur, with possibly fatal consequences if there is no prompt intervention [50,51]. During the course of our study, we did not encounter any of the abovementioned ADRs in our patient cohort. To date, our findings with regards to its use in childhood brain tumors concur with previous studies in the literature [8,9,10,52].

### 4.3. Use of Intraoperative Na-Fl: Institutional Reflections

In congruency with relevant publications, we report that that Na-Fl is well-tolerated by our patient population [8,9,52,53]. During the study period, no adverse side effects or complications were encountered. As an additional adjunct, it can be seamlessly integrated into the current neurosurgical workflow. In addition, its use complements other neurosurgical adjuncts that are used simultaneously. Overall, Na-Fl has favorable points: firstly, it is easy to prepare and administer at the beginning of surgery; next, most lesions of interest fluoresce to guide intraoperative surgical decision-making; and finally, it can be used for most pediatric brain tumors recruited in our study. Separately, it can be used either as a standalone adjunct or in combination with others. However, we observe some limitations as an intraoperative adjunct. Firstly, our experience with the use of Na-Fl fluorescence towards the end of resection is reported to be equivocal for distinct brain tumor demarcation. In addition, both cases of radiation-induced HGGs do not show detectable fluorescence in the operated lesion. Here, we are uncertain if our study’s dose of 2 mg/kg may be too low for some of the cases; and specifically for the two radiation-induced HGGs, if the tumors’ underlying biology has any bearing on our observations. Hence, we believe that more patient data and further optimization of our current Na-Fl doses as part of our ongoing work are required in order to draw objective conclusions.

### 4.4. Study Critique and Future Directions

The authors acknowledge that there are limitations of this study that should be highlighted. First and foremost, it is limited by its retrospective design and modest sample size. In addition, the method to assess the degree of Na-Fl fluorescence is based on a qualitative evaluation that is subject to the individual neurosurgeon. We are aware this is an imperfect approach, with its innate bias. Nevertheless, there is currently no established scoring system to measure the extent of intraoperative Na-Fl in real time. To circumvent this issue, the use of an image analysis platform to quantify the degree of fluorescence objectively has been described by a recent study [52]. We are investigating the use of a similar platform as part of our ongoing work.

At this stage, areas that require optimization include the usage in children less than 3 years old and a suitable dose of Na-Fl in our local pediatric population. This is because our study’s dosing regimen is currently more conservative that other reports in the literature [52]. Moving forward, there is certainly consideration to increase to a higher dose of Na-Fl prospectively for our study. In addition, we note there is variability in the types of neurosurgical procedures (i.e., burrhole versus craniotomy), anesthesia preparation after induction (such as setting of arterial and central lines, brain monitoring devices and so forth), setting up of neuronavigation and, for some cases, insertion of IONM probes. All of these contribute to differences in the timing between anesthesia induction and dura opening to confirm presence of Na-Fl fluorescence. At this point, we are still collecting data to determine the best timing between administration of Na-Fl and dura exposure. Nevertheless, this preliminary case series reports our initial experience with intraoperative Na-Fl is safe to be extrapolated to a larger cohort of our local pediatric patients for a prospective study. For now, we hope our findings add to the growing body of literature for the use of Na-Fl in childhood neurooncology, keeping in view the potential for future meta-analysis studies. In the meantime, we are cognizant that its implementation for routine clinical use remains a work in progress. 

## 5. Conclusions

Overall, preliminary findings from our pilot study demonstrate that Na-Fl is safe and reliable in our local context. However, ongoing work needs to address its feasibility in children less than 3 years old and ascertain if the underlying biology of selected brain tumors affects its efficacy. Furthermore, the optimal dose of Na-Fl in children, timing of administration in relation to dura opening and objective assessment of the degree of intraoperative fluorescence are factors that need to be addressed as part of our future undertaking. In the meantime, we strongly advocate global, collaborative efforts in the continued development of good operative adjuncts for pediatric neurooncology.

## Figures and Tables

**Table 1:** Patient demographics and variables. Note: values are n (%) unless stated otherwise.

Parameter	Value (%)
**Total number of patients**	21 (100)
**Age (years)**	12.1 ± 5.3 (mean ± SD)
**Gender**	
Male	13 (61.9)
Female	8 (38.1)
**Type of surgery**	
Biopsy	6 (28.6)
Resection	15 (71.4)
**Histopathological diagnosis ^1^**	
Non-neoplastic lesion (cavernoma)	1 (4.8)
Neoplastic lesions	20 (95.2)
*Low grade glioma*	6 (28.6)
*Hemispheric high grade glioma*	6 (28.6)
*Medulloblastoma*	2 (9.5)
*Diffuse midline glioma (H3K27M-altered)—1 thalamus and 2 brainstem*	3 (14.3)
*Craniopharyngioma*	1 (4.8)
*Choroid plexus carcinoma*	1 (4.8)
*Primary intracranial malignant melanoma*	1 (4.8)
**Adverse side effects from Na-Fl**	
Yes	0 (0)
No	21 (100)
**Score for Na-Fl fluorescence**	
0	3 (14.3)
1	0 (0)
2	5 (23.8)
3	13 (61.9)
**Concurrent operative adjuncts**	
DTI imaging	13 (61.9)
iMRI operating theatre	1 (4.8)
IONM	3 (14.3)
Transtubular system	3 (14.3)
NIL	6 (28.6)
**Location of lesion**	
Suprasellar	2 (9.5)
Frontal	2 (9.5)
Temporal	1 (4.8)
Parietal	4 (19.1)
Occipital	1 (4.8)
Thalamic	4 (19.1)
Intraventricular (frontal horn)	1 (4.8)
Posterior fossa	4 (19.1)
Brainstem	2 (9.5)

^1^ Histopathological diagnosis corresponds to the relevant WHO classification during recruitment period [14,15].

## Data Availability

Not applicable.
